# Clinical Outcomes of Rotational Atherectomy in Heavily Calcified Lesions: Evidence From the Largest Cardiac Center in Thailand

**DOI:** 10.5334/gh.1162

**Published:** 2022-10-25

**Authors:** Korakoth Towashiraporn, Rungroj Krittayaphong, Damras Tresukosol, Rewat Phankingthongkum, Wiwun Tungsubutra, Nattawut Wongpraparut, Narathip Chunhamaneewat, Asa Phichaphop, Pariya Panchavinnin, Treenet Reanthong, Chunhakasem Chotinaiwattarakul

**Affiliations:** 1 Her Majesty Cardiac Center, Faculty of Medicine Siriraj Hospital, Mahidol University, Bangkok, Thailand; 2 Department of Medicine, Faculty of Medicine Siriraj Hospital, Mahidol University, Bangkok, Thailand

**Keywords:** Debulking the calcified lesion, percutaneous coronary intervention, major adverse cardiovascular and cerebrovascular events

## Abstract

**Background:**

Evidence regarding the clinical outcomes of rotational atherectomy (RA) in middle-income countries is limited. We analyzed the clinical outcomes of patients with heavily calcified coronary lesions who underwent RA-assisted percutaneous coronary intervention (PCI) and explored the risks for developing major adverse cardiovascular and cerebrovascular events (MACCE).

**Methods:**

This is a single-center, retrospective cohort analysis that enrolled consecutive patients who underwent RA-assisted PCI at the largest tertiary hospital in Thailand. The primary endpoint is the incidence of MACCE during the first-year follow-up. MACCE consists of cardiac death, ischemic stroke, definite stent thrombosis, target lesion revascularization, and target vessel revascularization.

**Results:**

From January 2015 to December 2018, 616 patients (663 lesions) were enrolled. The mean age was 72.8 ± 9.7 years, 292 (47.4%) patients were female and 523 (84.9%) completed one-year follow-up. Drug-eluting stents were deployed in 606 (91.4%) lesions. The RA success rate – defined as when the operator successfully passed the burr across the target lesion – was 99.4% and the angiographic success rate was 94.8%. 130 (21.4%) procedures developed periprocedural complications. The cumulative MACCE rate at 30-days was 1.5% and at 1-year was 6.3%. The in-hospital mortality rate was 1.1% and the cardiac death rate was 1.6%. Independent risk factors for developing MACCE included the use of an intra-aortic balloon pump (hazard ratio [HR] 3.96, 95% confidence interval [CI] 1.54–10.21; P = 0.004), a history coronary artery bypass graft (HR 2.30, 95% CI 1.01–5.25; P = 0.048), and increased serum creatinine (HR 1.16, 95% CI 1.04–1.30; P = 0.008).

**Conclusions:**

RA is an effective revascularization technique for heavily calcified lesions. This study demonstrates a high success rate and good short- to intermediate-term results of RA-assisted PCI in middle-income countries which are similar to high-income countries. Nevertheless, the rate of periprocedural complications remains high.

## Introduction

Approximately 20% of patients who undergo percutaneous coronary intervention (PCI) have severe calcified coronary artery disease (CAD) [1]. Heavy calcification causes PCI to be more challenging as the calcific plaque prevents the coronary balloon from fully dilating during lesion preparation and impedes stent delivery. As a result, calcified plaque leads to suboptimal PCI results such as stent underexpansion and stent mal-apposition [2,3]. In addition, heavily calcified lesions are associated with long-term stent-related complications such as in-stent restenosis and stent thrombosis [4]. Consequently, coronary calcification adversely impacts clinical outcomes following PCI [5,6,7].

Rotational atherectomy (RA) is a technique that uses a dedicated diamond-coated burr to debulk the calcified lesions [8]. RA ablates the severely calcified plaque, enhancing optimal stent expansion and accommodating stent delivery [9,10]. As a result, RA improves the immediate procedural success rate and long-term clinical outcomes in patients with heavily calcified lesions [11].

There is limited evidence regarding the clinical outcomes of RA in middle-income countries. Therefore, we conducted a single-center, retrospective cohort analysis to investigate the short- and mid-term clinical outcomes of patients who were undergoing RA-assisted PCI. We also explored potential independent risk factors for major adverse cardiovascular and cerebrovascular events (MACCE).

## Methods

### Study designs and populations

We retrospectively enrolled all patients whom the operator decided to perform RA-assisted PCI, regardless of the RA outcome. The extent of coronary artery stenosis was assessed either by visual estimation, physiologic study, or intravascular imaging guidance. All RA procedures were performed at Her Majesty Cardiac Center, Faculty of Medicine Siriraj Hospital, Mahidol University, Bangkok, Thailand – a middle-income country in Southeast Asia – from January 2015 to December 2018. We divided the patients into a MACCE group and a MACCE-free group according to the occurrence of MACCE at the one-year follow-up. Two investigators manually extracted the relevant data from the electronic database into the dedicated case record form. The Siriraj Institutional Ethical Review Board of the Faculty of Medicine Siriraj Hospital, Mahidol University approved this cohort study (Protocol number 259/2562[EC2]).

### Rotational atherectomy

All RA procedures were performed using the Rotablator^TM^ system (Boston Scientific, Marlborough, MA, USA) by experienced operators following the standard recommendations [9,12]. The periprocedural medications and antithrombotic regimens were guided by standard clinical practice guidelines [13,14]. The indication for PCI, vascular access route, PCI technique, PCI strategy, the options of coronary stents, the determination to perform RA, RA burr size and amount, the timing of each run of RA, RA speed, and the total number of RA runs were at the operators’ discretion.

### Study outcomes

The primary objective was to measure the cumulative incidence of MACCE at the one-year follow-up visit. The secondary objective was to identify the independent risk factors for developing MACCE. MACCE consists of cardiac death, ischemic stroke, definite stent thrombosis, target lesion revascularization (TLR), and target vessel revascularization (TVR) during the follow-up period.

### Definitions

Cardiac death is defined as death caused by acute coronary syndrome (ACS) or heart failure. Ischemic stroke is defined as the presence of new neurological deficits lasting longer than 24 hours with any evidence of ischemia from any imaging modality. Definite stent thrombosis is defined as described elsewhere [15]. TLR is defined as any repeat revascularization at the target lesion which includes the 5-mm margin from the stent edge [16]. TVR is defined as any repeat revascularization of the target vessel [16]. Periprocedural myocardial infarction (MI) is defined as the absolute rise in serum creatinine kinase (CK-MB) of more than ten times the upper normal limit as described by the Society for Cardiovascular Angiography and Interventions [17]. No reflow phenomenon is defined as markedly limited coronary blood flow despite minimal residual stenosis, or no flow-limiting dissection at the target lesion. Contrast-induced nephropathy is defined as an absolute increase in serum creatinine (Cr) 0.5 mg/dl or more, or an increase in serum Cr at least 25% from baseline [18]. Angiographic success is defined as the achievement of residual stenosis not more than 20% after stent implantation. PCI procedural success is defined as angiographic success without major in-hospital adverse cardiac events [19]. RA procedural success is defined as when the operator could pass the burr across the target lesion. Intraprocedural complications consisted of flow-limiting coronary artery dissection after RA, coronary artery perforation, cardiac tamponade, no-reflow phenomenon required treatment, and burr entrapment. Periprocedural complications consisted of in-hospital death, in-hospital stroke, acute stent thrombosis, periprocedural MI, hemorrhage required blood transfusion, intracranial hemorrhage, fatal bleeding, vascular complications, and contrast-induced nephropathy.

### Statistical analysis and sample size calculation

Categorical data were presented as percentages and frequency. Quantitative variables with normal distribution were presented as mean ± standard deviation (SD), while skewness variables were demonstrated as median, minimum, and maximum values. We used the Chi-squared or Fisher’s exact test to compare categorical data and used the independent *t*-test or Mann-Whitney *U* test to compare the differences between continuous data. The cumulative MACCE was presented with a Kaplan-Meier curve and compared using Cox regression analysis. Cox’s proportional hazards regression model was used to identify the independent risk factors for developing MACCE and presented as hazard ratio (HR) and 95% confidence interval (95% CI). A p-value less than 0.05 was considered statistically significant. We used SPSS version 18 (SPSS Inc., Chicago, IL) to analyze the data.

To estimate the sample size, we used the estimation proportion of the one-group method. First, we estimated the cumulative incidence of MACCE at the one-year follow-up visit to be 16.3% [20]. Then, we prescribed the standard error of measurement at 20% and the Z-value at 1.96 for the 95% CI. The calculated sample size was 482 patients.

## Results

### Baseline clinical characteristics

**Figure 1:**
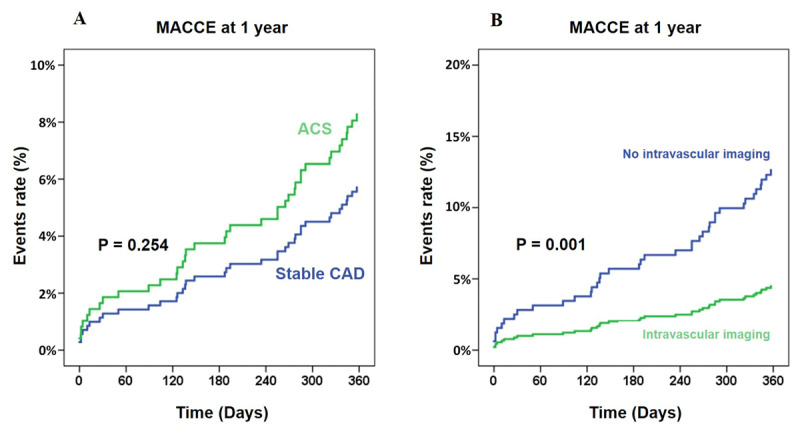
Impact of the clinical presentation and the use of intravascular imaging on the occurrence of MACCE. **(A)** The clinical presentation of the patients. ACS was the combination of non-ST elevation ACS and ST-segment elevation myocardial infarction. **(B)** Intravascular imaging consisted of intravascular ultrasound or optical coherence tomography. ACS – acute coronary syndrome; CAD – coronary artery disease; MACCE – major adverse cardiovascular and cerebrovascular events.

We enrolled 616 patients (663 lesions). The mean age of the cohort was 72.8 ± 9.7 years, 292 (47.4%) patients were female, 576 (93.5%) had hypertension, and 467 (75.8%) had dyslipidemia (Table 1). Most of the patients presented with stable CAD which had a non-significant difference in the incidence of MACCE compared to ACS patients (P = 0.254) (Figure 1). Among 367 patients with left ventricular ejection fraction (LVEF) data, the median LVEF was 60.00% (Interquartile range 40–70%). The mean serum Cr was 1.9 mg/dl, indicating mildly impaired renal function. The Cr level was statistically significantly higher (P = 0.048) and the hemoglobin level was statistically significantly lower (P < 0.001) in the MACCE group.

**Table 1:** Baseline clinical characteristics.


BASELINE CLINICAL CHARACTERISTICS	ALL (n =616)	MACCE GROUP (n =39)	MACCE-FREE GROUP (n =577)	p-VALUE

Age (year) – mean ± SD	72.81 ± 9.71	70.62 ± 9.48	72.96 ± 9.72	0.145

Sex (Female) – n (%)	292 (47.4%)	23 (59.0%)	269 (46.6%)	0.135

BMI (kg/m^2^) – mean ± SD	24.5 ± 4.2	25.1 ± 4.0	24.4 ± 4.2	0.360

Hypertension – n (%)	576 (93.5%)	38 (97.6%)	535 (93.2%)	0.502

Cerebrovascular disease – n (%)	30 (4.9%)	3 (7.7%)	27 (4.7%)	0.428

Diabetes require insulin therapy – n (%)	7 (1.1%)	0 (0.0%)	7 (1.2%)	0.489

Current or ex-smoker – n (%)	68 (11.0%)	2 (5.1%)	66 (11.4%)	0.297

Dyslipidemia – n (%)	467 (75.8%)	29 (74.4%)	438 (75.9%)	0.827

CKD required hemodialysis – n (%)	74 (12.0%)	10 (25.6%)	64 (11.1%)	**0.018**

History of previous ACS – n (%)	118 (19.2%)	6 (15.4%)	112 (19.4%)	0.536

History of previous CABG – n (%)	49 (8.0%)	7 (17.9%)	42 (7.3%)	**0.028**

History of previous PCI – n (%)	252 (40.9%)	14 (35.9%)	238 (41.2%)	0.511

LVEF – mean ± SD ^1^	55.59 ± 17.41	50.88 ± 16.18	55.94 ± 17.47	0.161

Hemoglobin level (g/dL) – mean ± SD ^2^	11.72 ± 1.82	10.35 ± 2.25	11.82 ± 1.75	**<0.001**

Creatinine level (mg/dL) – mean ± SD ^3^	1.90 ± 2.05	2.76 ± 2.75	1.84 ± 1.97	**0.048**

Indication stable CAD – n (%)	412 (66.9%)	23 (59.0%)	389 (67.4%)	0.278

Indication NSTE-ACS – n (%)	187 (30.4%)	14 (35.9%)	173 (30.0%)	0.437

Indication STEMI – n (%)	16 (2.6%)	2 (5.1%)	14 (2.4%)	0.269

Total lesion per patient – mean ± SD	1.08 ± 0.27	1.07 ± 0.26	1.08 ± 0.27	0.763


** Bold indicated statistically significant. 1. 40% of LVEF data were missing 2. 18.3% of serum Hb were imputed. 3. 14.3% of serum Cr were imputed. ACS – acute coronary syndrome; BMI – body mass index; CABG – coronary artery bypass graft; CKD – chronic kidney disease; LVEF – left ventricular ejection fraction; MACCE – major adverse cardiovascular and cerebrovascular events; NSTE-ACS- non-ST elevation acute coronary syndrome; PCI – percutaneous coronary intervention; SD – standard deviation; STEMI – ST-segment elevation myocardial infarction.

### Angiographic and procedural characteristics

Four hundred and forty-five (72.2%) of RA procedures were performed via the femoral approach (Table 2). Six hundred and fifty-two (98.3%) of the lesions were severely calcified and classified as type B2 or type C according to the American College of Cardiology and the American Heart Association system [21]. Forty-six (7.5%) lesions had chronic total occlusion lesions and 194 (31.5%) procedures involved bifurcation lesions. The two most common primary coronary de novo lesions involved the left anterior descending artery (372 lesions; 56.1%) and the right coronary artery (167 lesions; 25.2%). Sixty-four lesions (9.7%) involved the left main coronary artery. Balloon predilatation was performed in 126 (19%) of RA procedures, and balloon postdilatation was performed in 569 (85.8%) procedures. The operators used intravascular imaging guidance for PCI in 455 cases (73.9%), which was significantly higher in the MACCE-free group (75.4% versus 51.3%, P = 0.001).

**Table 2:** Angiographic and procedural characteristics.


CHARACTERISTICS	ALL (n = 663)	MACCE GROUP (n = 43)	MACCE-FREE GROUP (n = 620)	p-VALUE

**Angiographic characteristics**				

Thrombus (n) – n (%)	2 (0.3%)	0 (0.0%)	2 (0.3%)	0.713

Ostial lesion (n) – n (%)	113 (18.3%)	10 (25.6%)	103 (17.9%)	0.224

Chronic total occlusion (n) – n (%)	46 (7.5%)	2 (5.1%)	44 (7.6%)	0.759

Bifurcation (n) – n (%)	194 (31.5%)	11 (28.2%)	183 (31.7%)	0.648

Visible severe calcified coronary artery – n (%)	579 (94.0%)	37 (94.9%)	542 (93.9%)	0.811

**Procedural characteristics**				

Femoral access (n) – n (%)	445 (72.2%)	30 (76.9%)	415 (71.9%)	0.582

Target vessel – n (%)				0.330

LM to LAD or LM to LCX	64 (9.7%)	3 (7.0%)	61 (9.8%)	

LAD	372 (56.1%)	19 (44.2%)	353 (56.9%)	

LCX	59 (8.9%)	6 (14.0%)	53 (8.5%)	

RCA	167 (25.2%)	15 (34.9%)	152 (24.5%)	

Other	1 (0.2%)	0 (0.0%)	1 (0.2%)	

Target lesion B2 or C – n (%)	652 (98.3%)	43 (100%)	609 (98.2%)	0.378

Balloon dilate before RA – n (%)	126 (19.0%)	8 (18.6%)	118 (19.0%)	0.945

Post dilate after RA – n (%)	569 (85.8%)	38 (88.4%)	531 (85.6%)	0.620

Maximum diameter of postdilatation balloon (mm.) – mean ± SD	3.16 ± 0.56	3.18 ± 0.50	3.16 ± 0.56	0.803

Maximum pressure of postdilatation balloon (ATM.) – mean ± SD	20.22 ± 5.10	21.26 ± 6.00	20.14 ± 5.68	0.243

Maximum Burr size (mm.) – mean ± SD	1.55 ± 0.19	1.52 ± 0.19	1.55 ± 0.18	0.372

Use of laser – n (%)	2 (0.3%)	1 (2.3%)	1 (0.2%)	0.126

Successful RA – n (%)	659 (99.4%)	43 (100%)	616 (99.4%)	0.597

Number of burr(s) (n)				0.959

1 – n (%)	542 (81.8%)	36 (83.7%)	506 (81.7%)	

2 – n (%)	119 (17.9%)	7 (16.3%)	112 (18.1%)	

Maximum rotational speed x 10000 RPM – mean ± SD	184.88 ± 18.28	189.30 ± 11.36	184.57 ± 18.63	0.101

Intravenous pacemaker backup – n (%)	50 (7.5%)	5 (11.6%)	45 (7.3%)	0.362

Intra-aortic balloon pump – n (%)	26 (3.9%)	5 (11.6%)	21 (3.4%)	**0.021**

Stent deployment – n (%)	632 (95.3%)	41 (95.3%)	591 (95.3%)	0.994

POBA only – n (%)	23 (3.5%)	0 (0.0%)	23 (3.7%)	0.390

Drug-eluting stent – n (%)	606 (91.4%)	41 (95.3%)	565 (91.2%)	0.582

Diameter of the largest stent (mm.) – mean ± SD	3.18 ± 0.50	3.25 ± 0.52	3.17 ± 0.50	0.342

Number of stent(s) – n (%)				0.424

0	30 (4.5%)	2 (4.7%)	28 (4.5%)	

1	269 (40.6%)	16 (37.2%)	253 (40.8%)	

2	249 (37.6%)	13 (30.2%)	236 (38.1%)	

3	90 (13.6%)	9 (20.9%)	81 (13.1%)	

4	25 (3.8%)	3 (7.0%)	212 (3.5%)	

Maximum stent length (mm.) – mean ± SD	26.68 ± 7.71	25.54 ± 6.62	26.73 ± 7.77	0.327

Total stent length in target vessel – mean ± SD	41.51 ± 19.18	43.24 ± 21.24	41.39 ± 19.05	0.550

Maximum burr size – n (%)				0.882

1.25	110 (17.9%)	23 (20.0%)	87 (17.4%)	

1.50	297 (48.4%)	56 (48.7%)	241 (48.3%)	

1.75	196 (31.9%)	35 (30.4%)	161 (32.3%)	

2.00	10 (1.6%)	1 (0.9%)	9 (1.8%)	

2.25	1 (0.2%)	0 (0.0%)	1 (0.2%)	

Intravascular imaging – n (%)	455 (73.9%)	20 (51.3%)	435 (75.4%)	**0.001**

Procedural success – n (%)	481 (78.1%)	20 (51.3%)	461 (79.9%)	**<0.001**

Angiographic success – n (%)	584 (94.8%)	33 (92.3%)	548 (95.0%)	0.447

Total procedural time (min) – mean ± SD	91.43 ± 45.49	110.36 ± 55.10	90.15 ± 44.53	**0.007**

Total fluoroscopic time (min) – mean ± SD	35.16 ± 17.63	40.86 ± 16.31	34.78 ± 17.67	**0.037**

Total contrast volume (ml) – mean ± SD	177.24 ± 74.65	175.36 ± 77.68	177.37 ± 74.50	0.871


** Bold indicated statistically significant. ATM – atmospheric pressure; LAD – left anterior descending coronary artery; LCX – left circumflex coronary artery; LM – left main coronary artery; POBA – plain balloon angioplasty; RA – rotational atherectomy; RCA – right coronary artery; RPM – revolutions per minute.

Six hundred and fifty-nine (99.4%) procedures were successfully performed, and four procedures failed as the burr was unable to cross the culprit lesion. Angiographic success was achieved in 584 (94.8%) procedures. The two most commonly used burr sizes were 1.5 mm. (48.4%) and 1.75 mm. (31.39%). The maximum burr size used (P = 0.372) and the maximum RA speed (P = 0.101) were similar between groups. Coronary stents were deployed after completing RA in 632 (95.3%) procedures, and drug-eluting stents were used in 606 (91.4%) procedures. The mean stent length was 41.51 ± 19.18 mm. In 269 (40.6%) cases, only one coronary stent was used. An intravenous pacemaker was used in 50 (7.5%) procedures. Twenty-six cases (3.9%) used the intra-aortic balloon pump (IABP) for hemodynamic support during RA. The fluoroscopic time (P = 0.037) and procedural time (P = 0.007) were significantly longer in the MACCE-group. The use of intravascular imaging guidance had a significantly lower rate of MACCE (P = 0.001) (Figure 1).

### Intraprocedural and periprocedural complications

**Figure 2:**
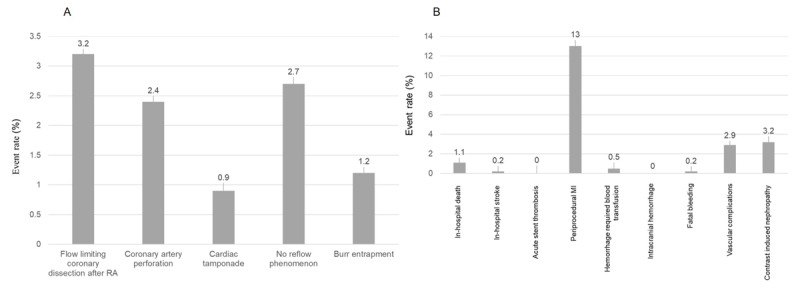
Intraprocedural and periprocedural complications. **(A)** intraprocedural complications that occurred during RA. **(B)** periprocedural complications post-RA. MI – myocardial infarction; RA – rotational atherectomy.

One hundred and thirty (19.6%) procedures developed periprocedural complications (Figure 2). The most common periprocedural complication was periprocedural MI (80 procedures; 13%). Coronary perforation occurred in 16 procedures (2.6%), and six (37.5%) of these developed cardiac tamponade. Seven cases of coronary perforation (43.8%) were treated with stent graft and two cases underwent emergency coronary artery bypass graft (CABG). Unfortunately, this catastrophic complication led to two in-hospital deaths. The incidence of burr entrapment occurred in eight (1.21%) cases. No acute stent thrombosis occurred.

### Clinical outcomes

**Figure 3:**
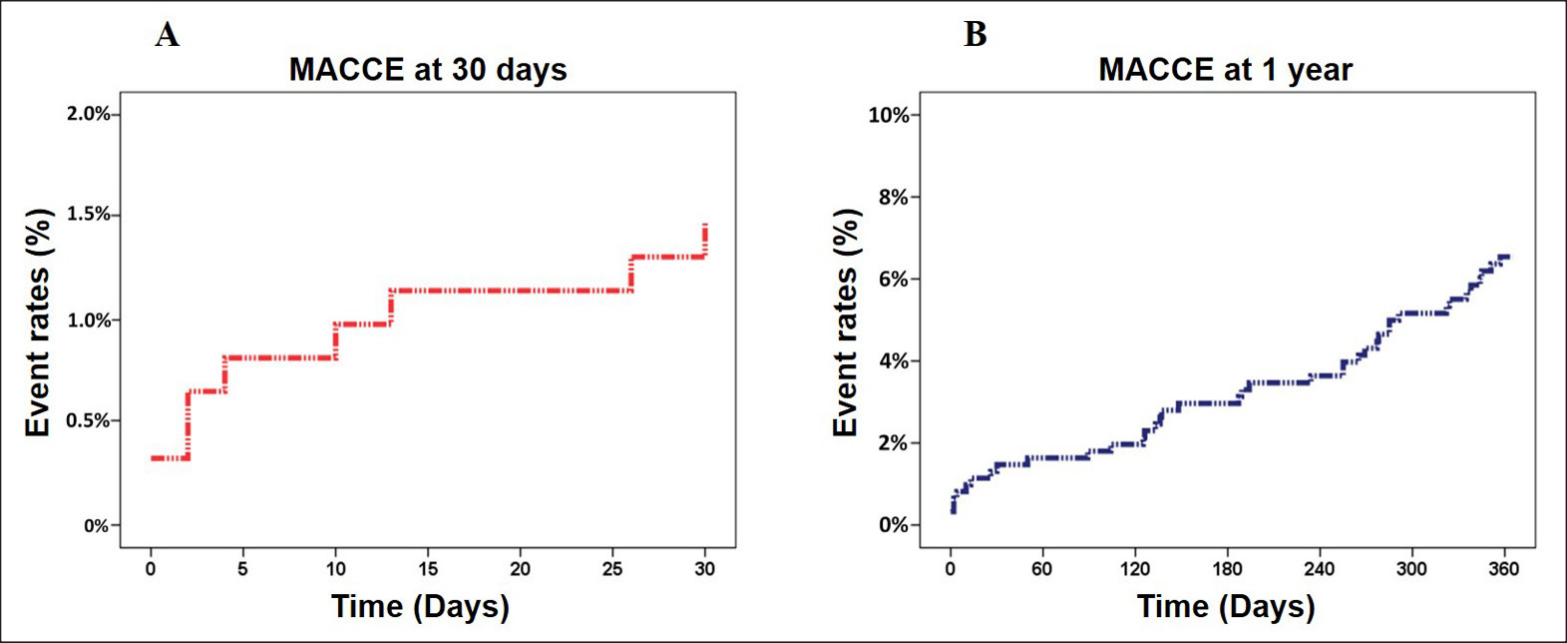
Major adverse cardiovascular and cerebrovascular events (MACCE) during follow-up. **(A)** MACCE during 30-days follow-up. **(B)** MACCE during one-year follow-up.

Five hundred and twenty-three patients (84.9%) completed one-year follow-ups. The incidence of all-cause death at the one-year follow-up was 3.6% (22 patients). The cumulative MACCE rate was 1.5% at 30-days and 6.3% at one-year (Figure 3). The rate of cardiac death was 1.0% at 30-days and 1.6% at one-year. The incidence of definite stent thrombosis gradually increased from 0.5% at 30-days to 2.8% at one-year. The incidence of stent-related complications (TLR and TVR) markedly increased from 0.3% to 3.6% at 30-days to 0.5% to 4.7% at the one-year follow-up. No ischemic stroke occurred during the follow-up period (Table 3).

**Table 3:** Primary adverse outcomes during the follow-up period.


ADVERSE OUTCOMES	TOTAL, n (%)

**30-day MACCE – n (%)**	9 (1.5%)

Cardiac death	6 (1.0%)

Ischemic stroke	0 (0.0%)

Definite stent thrombosis	(0.5%)

TLR	2 (0.3%)

TVR	3 (0.5%)

**1-year MACCE – n (%)**	39 (6.3%)

Cardiac death	10 (1.6%)

Ischemic stroke	0 (0.0%)

Definite stent thrombosis	17 (2.8%)

TLR	22 (3.6%)

TVR	29 (4.7%)


MACCE – major adverse cardiovascular and cerebrovascular events; TLR – target lesion revascularization; TVR – target vessel revascularization.

### Predictors of MACCE

**Figure 4:**
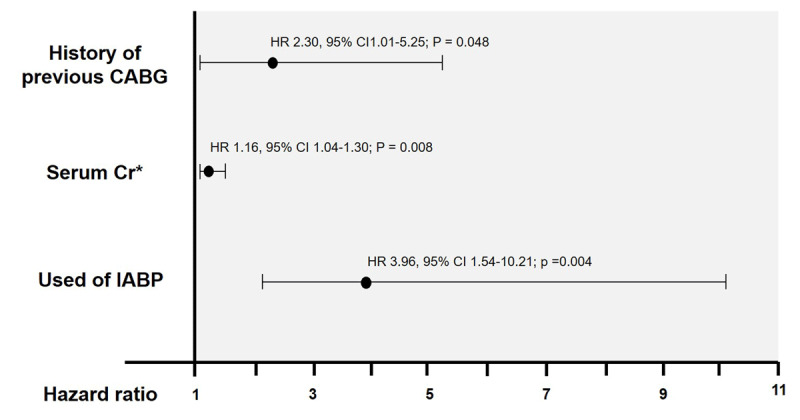
Multivariate analysis adjusted for the independent risk of developing MACCE. CABG – coronary artery bypass graft; 95% CI – 95% confidence interval; HR – hazard ratio. * 14.3% of serum Cr values were imputed.

A history of previous CABG (HR 2.30, 95% CI 1.01–5.25; P = 0.048), the use of IABP (HR 3.96, 95% CI 1.54–10.21; P = 0.048), and increased serum Cr from the reference value at 1 mg/dl (HR 1.16, 95% CI 1.04–1.30; P = 0.008) were associated with MACCE at the one-year follow-up. (Figure 4).

## Discussion

Our results support that RA is a feasible revascularization technique for heavily calcified lesions that provides satisfactory immediate PCI results and good, short- to mid-term clinical outcomes. However, periprocedural complications were high. Intravascular imaging guidance for RA-assisted PCI may reduce the occurrence of MACCE. Impaired renal function is associated with adverse clinical outcomes in patients undergoing RA-assisted PCI. We observed high RA and angiographic success rates (99.4% and 94.8%, respectively) that were similar to other published registries investigated in high-income countries [22,23,24,25]. Together, our analysis suggests that RA-assisted PCI is an effective strategy for heavily calcified lesions in daily practice.

The majority (66.9%) of our participants had stable CAD. We found that ACS patients who underwent RA had rates of MACCE comparable with those with RA with stable CAD (P = 0.254) (Figure 1). This is in contrast to a multi-center registry from Italy that reported higher adverse cardiac events in ACS patients who underwent RA compared to RA in stable CAD [26]. The Euro4C registry also reported that ACS is an independent predictor for developing MACCE post-RA [27]. The most common vascular access site in our study was the femoral route (72.2%). This is similar to the ROTATE registry in Japan that enrolled subjects between 2002 to 2013 and reported that the femoral route was used in 71.6% of cases [28]. This is in contrast to other published registries [23,27] that reported preferential vascular access via the radial approach. We observed a vascular complications rate of 2.9%, similar to the 2.5% rate reported in a registry of 103,070 PCI patients (98.9% femoral approach) [29].

Interestingly, our rate of intravascular imaging-guided PCI was markedly higher (73.9%) than the Euro4C registry (6.9%) [27]. This finding may explain the relatively low rate of cardiac death and stent-related complications (TLR and TVR) we observed compared to other published registries [22,23,24] (Table 4). In a meta-analysis that included 4,724 patients from nine randomized controlled trials, intravascular imaging-guided PCI significantly reduced the rate of cardiac death and coronary revascularization [30]. Our findings, therefore, support the use of intravascular imaging guidance during RA-assisted PCI. However, further randomized control trials are needed to conduct to confirm this hypothesis.

**Table 4:** Comparison of recently published rotational atherectomy registries that were conducted in Asia.


CLINICAL TRIAL	POPULATION	NUMBER OF LESIONS	ACS (%)	CENTER(S)	COUNTRY	ANGIOGRAPHIC SUCCESS (%)	FOLLOW UP DURATION	CARDIAC DEATH (%)	STENT THROMBOSIS (%)	TLR (%)	TVR (%)	STROKE (%)

Lee K et al. [22]	540	583	60.5%	Multi-center	Korea	96.4	1.5 years	6.9	1.2	8.2	9.8	2.0

Gao W et al. [23]	540	540	14.6%	Single-center	China	96.8	In-hospital	1.1	0	–	–	0

Okai I et al. [24]	1090	–	15%	Multi-center	Japan	96.2	3.8 years	10.9	1.4	17.7	23	5.1

Ray S et al. [25]	144	–	31.25%	Single-center	India	96.5	1 year	0.7	–	–	2.1	–

Our registry	616	663	33.1%	Single-center	Thailand	94.8	1 year	1.6	2.8	3.6	4.7	0


ACS – acute coronary syndrome; TLR – target lesion revascularization; TVR – target vessel revascularization.

The high rates of periprocedural complications we observed were mainly driven by the rate of periprocedural MI (13%). Nevertheless, the incidence of intraprocedural complications was acceptable. Our rate of periprocedural MI was higher than the Korea-ROCK registry which reported the rate of periprocedural MI at 7.9% [22] and Park DW, et al. that enrolled 23,604 patients from 11 clinical studies (7%) [31]. This may be explained by the high number of complex lesions, (98.3% were type B2 or C) in our study, and the criteria to diagnose periprocedural MI using the elevation of serum CK-MB that may overdiagnose [22]. In addition, calcified lesions and RA procedures enhance the risk of rising postprocedural serum CK-MB levels [32]. We observed that burr entrapment occurred in 1.2% of RA, which is higher than the 0.4% rate reported in a study by Nobuyoshi M, et al. [33]. The absolute amounts of contrast media used in our study were numerically lower than the ROCK registry (Mean contrast media 177 ml. versus 210 ml.). Nevertheless, the rate of contrast-induced nephropathy was similar (3.2% versus 3.3%) [22].

Our results demonstrate the impact of the serum Cr level on the occurrence of MACCE. We found that for each 1 mg/dl of serum Cr increase, the incidence of MACCE increased 1.16 times (HR 1.16, 95% CI 1.04–1.30; P = 0.008). The mean serum Cr in our study was relatively high (Cr 1.9 mg/dl.), which suggests that RA should be performed cautiously in the presence of renal insufficiency, as these patients are at high risk for developing MACCE. Patients who used IABP during RA and patients with a history of prior CABG were also at increased risk of MACCE. However, our finding was in contrast to evidence from the Euro4C registry that demonstrated a protective effect of prior CABG against the occurrence of adverse clinical outcomes [27].

Our non-randomized, retrospective study has limitations. The design leaves open the possibility that confounding factors and sources of bias [34], known and unknown, may have influenced our analysis. In this present study, several data are missing, for example, 40% of LVEF data are missing. We also impute 18.3% of serum Hb and 14.3% of serum Cr. Nevertheless, our findings reflect real-world data regarding the RA procedure in middle-income countries. Second, stent-related complications may have been underdiagnosed because controlled coronary angiography was not routinely performed. Third, our study does not have access to information regarding patients’ medication at discharge, specific lesion characteristics, and details of the coronary stents used. Finally, because this was a tertiary-referral center cohort, our findings may not be generalizable to other settings. In addition, our data were collected between 2015–2018, in the era that the shockwave intravascular lithotripsy, which was recently approved by the U.S. Food and Drug Administration for treating coronary calcification, was scantly used. However, this device is not currently available worldwide, especially in low- to middle-income countries.

## Conclusions

We conclude that RA in the DES-era is useful for treating heavily calcified coronary artery disease. This procedure in middle-income countries still provides a high procedural success rate and good short- to mid-term results similar to the results from high-income countries. Nevertheless, periprocedural complications – especially MI – were high. The use of an IABP during RA, a his tory of CABG, and increased serum Cr are strongly associated with MACCE. On the other hand, the use of intravascular imaging guidance for PCI potentially reduces the rates of adverse events.

## References

[1] Bourantas CV, Zhang YJ, Garg S, Iqbal J, Valgimigli M, Windecker S, et al. Prognostic implications of coronary calcification in patients with obstructive coronary artery disease treated by percutaneous coronary intervention: a patient-level pooled analysis of 7 contemporary stent trials. Heart. 2014; 100(15): 1158–64. DOI: 10.1136/heartjnl-2013-305180 24846971

[2] Henneke KH, Regar E, Konig A, Werner F, Klauss V, Metz J, et al. Impact of target lesion calcification on coronary stent expansion after rotational atherectomy. Am Heart J. 1999; 137(1): 93–9. DOI: 10.1016/S0002-8703(99)70463-1 9878940

[3] Kobayashi Y, Okura H, Kume T, Yamada R, Kobayashi Y, Fukuhara K, et al. Impact of target lesion coronary calcification on stent expansion. Circ J. 2014; 78(9): 2209–14. DOI: 10.1253/circj.CJ-14-0108 25017740

[4] Salazar C, Escaned J, Tirado G, Gonzalo N. Undilatable Calcific Coronary Stenosis Causing Stent Underexpansion and Late Stent Thrombosis: A Complex Scenario Successfully Managed With Intravascular Lithotripsy. JACC Cardiovasc Interv. 2019; 12(15): 1510–2. DOI: 10.1016/j.jcin.2019.02.010 30981572

[5] Sharma SK, Bolduan RW, Patel MR, Martinsen BJ, Azemi T, Giugliano G, et al. Impact of calcification on percutaneous coronary intervention: MACE-Trial 1-year results. Catheter Cardiovasc Interv. 2019; 94(2): 187–94. DOI: 10.1002/ccd.28099 30681262

[6] Lee MS, Yang T, Lasala J, Cox D. Impact of coronary artery calcification in percutaneous coronary intervention with paclitaxel-eluting stents: Two-year clinical outcomes of paclitaxel-eluting stents in patients from the ARRIVE program. Catheter Cardiovasc Interv. 2016; 88(6): 891–7. DOI: 10.1002/ccd.26395 26756859

[7] Zhang Y, Song L, Song Y, Xu LJ, Wang HH, Xu JJ, et al. Impact of coronary artery lesion calcification on the long-term outcome of patients with coronary heart disease after percutaneous coronary intervention. Zhonghua Xin Xue Guan Bing Za Zhi. 2019; 47(1): 34–41. DOI: 10.3760/cma.j.issn.0253-3758.2019.01.004 30669808

[8] Iannopollo G, Gallo F, Mangieri A, Laricchia A, Erriquez A, Tzanis G, et al. Tips and Tricks for Rotational Atherectomy. J Invasive Cardiol. 2019; 31(12): E376–E83. 31786529 10.25270/jic/19.3112.E376

[9] Sharma SK, Tomey MI, Teirstein PS, Kini AS, Reitman AB, Lee AC, et al. North American Expert Review of Rotational Atherectomy. Circ Cardiovasc Interv. 2019; 12(5): e007448. DOI: 10.1161/CIRCINTERVENTIONS.118.007448 31084239

[10] Stankovic G, Milasinovic D. Rotational Atherectomy in Clinical Practice: The Art of Tightrope Walking. Circ Cardiovasc Interv. 2016; 9(11). DOI: 10.1161/CIRCINTERVENTIONS.116.004571 27974433

[11] Otaki Y, Ashikaga T, Sasaoka T, Kurihara K, Yoshikawa S, Isobe M, et al. Long-term clinical outcomes of permanent-polymer everolimus-eluting stent implantation following rotational atherectomy for severely calcified de novo coronary lesions: Results of a 22-center study (Tokyo-MD PCI Study). Cardiovasc Revasc Med. 2019; 20(2): 120–5. DOI: 10.1016/j.carrev.2018.04.022 29861332

[12] Sakakura K, Ito Y, Shibata Y, Okamura A, Kashima Y, Nakamura S, et al. Clinical expert consensus document on rotational atherectomy from the Japanese association of cardiovascular intervention and therapeutics. Cardiovasc Interv Ther. 2021; 36(1): 1–18. DOI: 10.1007/s12928-020-00715-w 33079355 PMC7829233

[13] Neumann FJ, Sousa-Uva M, Ahlsson A, Alfonso F, Banning AP, Benedetto U, et al. ESC Scientific Document Group. 2018 ESC/EACTS Guidelines on myocardial revascularization. Eur Heart J. 2019 Jan 7; 40(2): 87–165. DOI: 10.1093/eurheartj/ehy39410.1093/eurheartj/ehy39430165437

[14] Levine GN, Bates ER, Blankenship JC, Bailey SR, Bittl JA, Cercek B, et al. 2011 ACCF/AHA/SCAI Guideline for Percutaneous Coronary Intervention: executive summary: a report of the American College of Cardiology Foundation/American Heart Association Task Force on Practice Guidelines and the Society for Cardiovascular Angiography and Interventions. Circulation. 2011; 124(23): 2574–609. DOI: 10.1161/CIR.0b013e31823a5596 22064598

[15] Cutlip DE, Windecker S, Mehran R, Boam A, Cohen DJ, van Es GA, et al. Clinical end points in coronary stent trials: a case for standardized definitions. Circulation. 2007; 115(17): 2344–51. DOI: 10.1161/CIRCULATIONAHA.106.685313 17470709

[16] Garcia-Garcia HM, McFadden EP, Farb A, Mehran R, Stone GW, Spertus J, et al. Standardized End Point Definitions for Coronary Intervention Trials: The Academic Research Consortium-2 Consensus Document. Circulation. 2018; 137(24): 2635–50. DOI: 10.1161/CIRCULATIONAHA.117.029289 29891620

[17] Moussa ID, Klein LW, Shah B, Mehran R, Mack MJ, Brilakis ES, et al. Consideration of a new definition of clinically relevant myocardial infarction after coronary revascularization: an expert consensus document from the Society for Cardiovascular Angiography and Interventions (SCAI). J Am Coll Cardiol. 2013; 62(17): 1563–70. DOI: 10.1016/j.jacc.2013.08.720 24135581 PMC3890321

[18] Mehran R, Nikolsky E. Contrast-induced nephropathy: definition, epidemiology, and patients at risk. Kidney Int Suppl. 2006; 69(100): S11–5. DOI: 10.1038/sj.ki.5000368 16612394

[19] Smith SC Jr., Dove JT, Jacobs AK, Kennedy JW, Kereiakes D, Kern MJ, et al. ACC/AHA guidelines for percutaneous coronary intervention (revision of the 1993 PTCA guidelines)-executive summary: a report of the American College of Cardiology/American Heart Association task force on practice guidelines (Committee to revise the 1993 guidelines for percutaneous transluminal coronary angioplasty) endorsed by the Society for Cardiac Angiography and Interventions. Circulation. 2001; 103(24): 3019–41. DOI: 10.1161/01.CIR.103.24.3019 11413094

[20] Cuenza LR, Jayme AC, Khe Sui JH. Clinical Outcomes of Patients Undergoing Rotational Atherectomy Followed by Drug-eluting Stent Implantation: A Single-center Real-world Experience. Heart Views. 2017; 18(4): 115–20. DOI: 10.4103/1995-705X.221231 29326773 PMC5755191

[21] Ellis SG, Vandormael MG, Cowley MJ, DiSciascio G, Deligonul U, Topol EJ, et al. Coronary morphologic and clinical determinants of procedural outcome with angioplasty for multivessel coronary disease. Implications for patient selection. Multivessel Angioplasty Prognosis Study Group. Circulation. 1990; 82(4): 1193–202. DOI: 10.1161/01.CIR.82.4.1193 2401060

[22] Lee K, Jung JH, Lee M, Kim DW, Park MW, Choi IJ, et al. Clinical Outcome of Rotational Atherectomy in Calcified Lesions in Korea-ROCK Registry. Medicina (Kaunas). 2021; 57(7). DOI: 10.3390/medicina57070694 PMC8303478 34356975

[23] Gao W, Chen Y, Yang H, Yao K, Ge J. Outcomes of rotational atherectomy for severely calcified coronary lesions: A single center 5-year experience. Catheter Cardiovasc Interv. 2021; 98(2): E254–E61. DOI: 10.1002/ccd.29740 33964182

[24] Okai I, Dohi T, Okazaki S, Jujo K, Nakashima M, Otsuki H, et al. Clinical Characteristics and Long-Term Outcomes of Rotational Atherectomy- J2T Multicenter Registry. Circ J. 2018; 82(2): 369–75. DOI: 10.1253/circj.CJ-17-0668 28931790

[25] Ray S, Bandyopadhyay S, Bhattacharjee P, Mukherjee P, Karmakar S, Mitra S, et al. Percutaneous coronary intervention of severely/moderately calcified coronary lesions using single-burr rotational atherectomy: A retrospective study. Anatol J Cardiol. 2021; 25(6): 395–401. DOI: 10.14744/AnatolJCardiol.2020.81335 34100726 PMC8210926

[26] Iannaccone M, Piazza F, Boccuzzi GG, D’Ascenzo F, Latib A, Pennacchi M, et al. ROTational AThErectomy in acute coronary syndrome: early and midterm outcomes from a multicentre registry. EuroIntervention. 2016; 12(12): 1457–64. DOI: 10.4244/EIJ-D-15-00485 27998837

[27] Bouisset F, Barbato E, Reczuch K, Dobrzycki S, Meyer-Gessner M, Bressollette E, et al. Clinical outcomes of PCI with rotational atherectomy: the European multicentre Euro4C registry. EuroIntervention. 2020; 16(4): e305–e12. DOI: 10.4244/EIJ-D-19-01129 32250249

[28] Kawamoto H, Latib A, Ruparelia N, Ielasi A, D’Ascenzo F, Pennacchi M, et al. In-hospital and midterm clinical outcomes of rotational atherectomy followed by stent implantation: the ROTATE multicentre registry. EuroIntervention. 2016; 12(12): 1448–56. DOI: 10.4244/EIJ-D-16-00386 27998836

[29] Grossman PM, Gurm HS, McNamara R, Lalonde T, Changezi H, Share D, et al. Percutaneous coronary intervention complications and guide catheter size: bigger is not better. JACC Cardiovasc Interv. 2009; 2(7): 636–44. DOI: 10.1016/j.jcin.2009.05.012 19628187

[30] Gao XF, Wang ZM, Wang F, Gu Y, Ge Z, Kong XQ, et al. Intravascular ultrasound guidance reduces cardiac death and coronary revascularization in patients undergoing drug-eluting stent implantation: results from a meta-analysis of 9 randomized trials and 4724 patients. Int J Cardiovasc Imaging. 2019; 35(2): 239–47. DOI: 10.1007/s10554-019-01555-3 30747368

[31] Park DW, Kim YH, Yun SC, Ahn JM, Lee JY, Kim WJ, et al. Frequency, causes, predictors, and clinical significance of peri-procedural myocardial infarction following percutaneous coronary intervention. Eur Heart J. 2013; 34(22): 1662–9. DOI: 10.1093/eurheartj/eht048 23404537

[32] Lansky AJ, Stone GW. Periprocedural myocardial infarction: prevalence, prognosis, and prevention. Circ Cardiovasc Interv. 2010; 3(6): 602–10. DOI: 10.1161/CIRCINTERVENTIONS.110.959080 21156928

[33] Nobuyoshi M. Experience is the best teacher. Catheter Cardiovasc Interv. 2000; 49(1): 85. DOI: 10.1002/(SICI)1522-726X(200001)49:1<85::AID-CCD19>3.0.CO;2-R 10627375

[34] Talari K, Goyal M. Retrospective studies – utility and caveats. J R Coll Physicians Edinb. 2020; 50(4): 398–402. DOI: 10.4997/JRCPE 33469615

